# Assessment of Patients’ Perceptions and Satisfaction With Urgent Care Services at King Saud Medical City, Riyadh, Saudi Arabia: A Cross-Sectional Study

**DOI:** 10.7759/cureus.109167

**Published:** 2026-05-19

**Authors:** Malak F Alsubie, Shrouq H Alkhaibari, Yasser S Aljaied, Suha S Algain, Bander S Alshehry

**Affiliations:** 1 Family Medicine, King Saud Medical City, Riyadh, SAU

**Keywords:** king saud medical city, patient perceptions, patient’s satisfaction, quality of care provided, riyadh first health care cluster, urgent care clinic

## Abstract

Background

Patient satisfaction is an important indicator of healthcare quality and patient-centered service delivery, particularly in urgent care settings where timely assessment, clear communication, appropriate triage, and safe discharge planning are essential. In Saudi Arabia, urgent care services have gained increasing importance as part of efforts to improve access, support patient flow, and reduce unnecessary emergency department utilization. However, evidence regarding patients’ perceptions and satisfaction with urgent care services in large tertiary healthcare institutions remains limited.

Objective

To assess patients’ perceptions and satisfaction with urgent care services at King Saud Medical City, Riyadh, Saudi Arabia, and to examine selected patient and service-related factors associated with urgent care experience outcomes.

Methods

A cross-sectional study was conducted between June 3, 2025, and January 25, 2026, among adult patients attending the urgent care clinic at King Saud Medical City. Eligible participants were adults aged 18 to 80 years who were triaged as Emergency Severity Index level 4 or 5 and were able to provide informed consent. Data were collected using a structured questionnaire assessing demographic characteristics, reason for visit, waiting time, overall satisfaction, satisfaction with waiting time, perceived quality of care, service quality indicators, follow-up information, and willingness to return for future care. Descriptive statistics and chi-square analyses were performed.

Results

A total of 200 patients were included. Male participants represented 110 patients (55.0%), and the mean age was 34.9 ± 12.8 years. The most common presenting complaints were musculoskeletal and minor orthopedic complaints, 51 (25.5%), followed by superficial soft-tissue injuries, 35 (17.5%), and gastrointestinal complaints, 34 (17.0%). Most patients were assessed within 30 minutes, 183 (91.5%). Overall satisfaction was high, with 183 patients (91.5%) reporting that they were satisfied or very satisfied, while 167 patients (83.5%) were satisfied or very satisfied with waiting time. Most patients reported that their concerns were addressed effectively, 193 (96.5%), clear instructions were provided, 195 (97.5%), and providers were knowledgeable and professional, 192 (96.0%). However, only 128 patients (64.0%) reported being informed about follow-up care. Overall quality of care rating was significantly associated with overall satisfaction (χ² = 15.67, p < 0.001). Waiting time duration was significantly associated with overall satisfaction (χ² = 13.59, p < 0.001) and willingness to return for future care (χ² = 12.19, p < 0.001). Age group was not significantly associated with satisfaction with waiting time (p = 0.435).

Conclusion

Patients attending the urgent care clinic at King Saud Medical City reported high levels of satisfaction, favorable perceived quality of care, and positive service quality indicators. Shorter waiting time and higher perceived quality of care were significantly associated with better patient experience outcomes, including overall satisfaction and willingness to return. Follow-up communication emerged as a potential area for improvement. Strengthening discharge counseling, safety-netting advice, and follow-up instructions may further enhance continuity of care and patient-centered urgent care delivery.

## Introduction

Patient satisfaction is an important measure of healthcare quality because it reflects how patients experience care from their own perspective, not only whether a clinical diagnosis or treatment was provided. It captures whether patients felt heard, respected, reassured, and managed in a timely and effective manner. This is particularly important in urgent care settings, where patients often present with sudden or unexpected health concerns and expect rapid assessment, appropriate triage, clear communication, and safe discharge instructions. In these encounters, satisfaction is shaped by several connected factors, including waiting time, accessibility, staff communication, perceived urgency, expectations of care, and the patient’s confidence that their immediate health concern has been properly addressed [1,2].

Urgent care services serve as a bridge between primary healthcare centers and emergency departments. They are designed for patients who require prompt medical attention, but whose conditions may not require the full resources of an emergency department. In Saudi Arabia, this model of care has gained increasing attention as part of national efforts to improve healthcare access, organize patient flow, and reduce unnecessary pressure on emergency services. Recently, the Ministry of Health emphasized the standardization of Urgent Care Centers by defining their operational pathways, staffing requirements, quality control measures, and service models across the Kingdom [3]. This reflects a broader national direction toward strengthening urgent care as a structured, accessible, and patient-centered component of the healthcare system.

In Riyadh, available evidence suggests that patients generally report positive experiences with urgent care services, although specific areas for improvement remain. A cross-sectional study conducted among patients attending urgent care clinics at Prince Sultan Military Medical City primary healthcare centers found favorable satisfaction levels, particularly regarding provider interactions and services. However, lower satisfaction was reported in relation to waiting time, parking availability, and patient involvement in decision-making [4]. These findings suggest that satisfaction in urgent care is not determined by clinical care alone, but by the patient’s entire journey through the service, from arrival and access to communication, participation in care, and discharge.

Related Saudi evidence from acute and ambulatory care settings further supports the importance of waiting time, communication, and service quality in shaping patient satisfaction. In a prospective study conducted at an emergency care center in central Saudi Arabia, Abolfotouh et al. identified waiting time as a key modifiable predictor of patient satisfaction, emphasizing the operational importance of timely patient flow in acute-care environments [5]. In primary healthcare settings, Alrasheedi et al. reported a significant association between wait times and patient satisfaction in Saudi Arabia, further supporting the role of service efficiency in patient experience [6]. At the national level, Almass et al. assessed patient satisfaction with quality of care in Saudi Arabia and highlighted the relevance of patient-centered service delivery in shaping satisfaction with healthcare services [7]. Similarly, a Saudi study of primary healthcare centers found that patients were generally satisfied with service quality, with provider-related care representing one of the strongest satisfaction domains [8].

Beyond Riyadh, however, published evidence specifically assessing patient satisfaction with urgent care services in other Saudi cities and the wider Middle East remains limited. Because of this scarcity of direct regional studies, international evidence from similar urgent and after-hours care models can provide useful context. In Canada, Howard et al. assessed patient satisfaction among family practice patients who sought care for urgent health problems and found that satisfaction was highest when care was provided by the patient’s own family physician or by an after-hours clinic affiliated with the physician’s practice [9]. Their findings emphasized that satisfaction in urgent care is influenced not only by rapid access but also by continuity of care, familiarity with the provider, and confidence in the care pathway [9].

Despite the growing role of urgent care services in Saudi Arabia, research in this area remains limited, particularly within large tertiary healthcare institutions. King Saud Medical City is a major tertiary healthcare institution within Riyadh First Health Cluster and serves a large and diverse patient population with varying clinical needs and expectations. Understanding patients’ perceptions and satisfaction with urgent care services in this setting is therefore essential to identify service strengths, recognize areas requiring improvement, and guide future patient-centered quality improvement initiatives. Therefore, this study aimed to assess patients’ perceptions and satisfaction with urgent care services at King Saud Medical City, Riyadh, Saudi Arabia.

Objectives

The objective of this study was to assess patients’ perceptions and satisfaction with urgent care services at King Saud Medical City, Riyadh, Saudi Arabia. Specifically, the study evaluated overall satisfaction, satisfaction with waiting time, perceived quality of care, and key service quality indicators, including whether patients felt their concerns were addressed, whether clear instructions were provided, whether healthcare providers were perceived as knowledgeable and professional, whether follow-up care was explained, and whether patients would return for future care. The study also aimed to describe the demographic profile of patients attending urgent care services, identify the main categories of presenting complaints, and evaluate operational performance through waiting time distribution and selected key performance indicators. Finally, the study examined factors associated with satisfaction with waiting time, including actual waiting time, reason for visit, and gender. By examining both patient satisfaction and service performance measures, this study sought to identify areas of strength and potential opportunities for improvement, particularly in relation to patient flow, communication, follow-up care, and overall patient-centered urgent care delivery.

## Materials and methods

Study design and setting

A cross-sectional study was conducted between June 3, 2025, and January 25, 2026, among adult patients attending the urgent care clinic at King Saud Medical City, Riyadh, Saudi Arabia. Ethical approval for this study was obtained from the Institutional Review Board at King Saud Medical City, Riyadh, Saudi Arabia. The study was approved as an exempt review under proposal reference number H1RI-01-May25-02, dated May 19, 2025. King Saud Medical City is a major tertiary healthcare institution within Riyadh First Health Cluster. The study was designed to provide a snapshot of patients’ perceptions and satisfaction with urgent care services, including waiting time, overall satisfaction, perceived quality of care, communication, follow-up information, and selected service quality indicators.

Study population

Eligible participants were adult patients aged 18 to 80 years who attended the urgent care clinic during the study period, were triaged as Emergency Severity Index (ESI) level 4 or 5, had completed their urgent care encounter, and were able to provide informed consent. The ESI is a five-level emergency department triage algorithm that categorizes patients according to clinical acuity and anticipated resource needs, ranging from level 1, which represents the most urgent cases, to level 5, which represents the least urgent cases [10]. Patients were excluded if they were younger than 18 years or older than 80 years, triaged as ESI level 1, 2, or 3, had cognitive impairment, or were unable to provide informed consent. 

Sample size and sampling procedure

A total of 200 eligible patients were included in the final analysis. The sample size was determined based on feasibility, patient flow, and the number of eligible patients who attended the urgent care clinic at King Saud Medical City and agreed to participate during the data collection period, which extended from June 3, 2025, to January 25, 2026. Although random probability sampling was not employed, a structured time-based convenience recruitment approach was used to improve sample diversity and better reflect routine urgent care utilization across different clinic hours. Data collection was conducted across different days of the week and across three clinic shifts: morning shift from 8:00 AM to 3:59 PM, evening shift from 4:00 PM to 11:59 PM, and overnight shift from 12:00 AM to 7:59 AM. During each data collection period, eligible patients attending the urgent care clinic were approached and invited to participate. This approach was intended to capture variation in patient flow, presenting complaints, and service experience across different working hours. Participation was voluntary, and verbal informed consent was obtained before enrollment. Patients who completed the questionnaire and met the eligibility criteria were included in the final analysis.

Data collection

During urgent care clinic visits, eligible patients were invited to complete a structured questionnaire after receiving care to ensure that their responses reflected the full visit experience. The questionnaire was developed specifically for this study by experienced physicians in family medicine to assess patients’ perceptions and satisfaction with urgent care services at King Saud Medical City. The questionnaire included demographic information, reason for visit, waiting time from triage to provider assessment, satisfaction with waiting time, overall satisfaction, perceived quality of care, communication quality, clarity of instructions, follow-up information, and willingness to return for future care. Satisfaction with waiting time and overall satisfaction were assessed using a five-point Likert scale. Waiting time was recorded in predefined categories, and reasons for visit were initially documented according to the presenting complaint and later recoded into system-based categories by a clinician to allow structured analysis. Prior to full data collection, the questionnaire was pilot tested on a small sample of 12 patients who were not included in the final study sample. Feedback from this pilot phase informed minor revisions to improve the clarity, content validity, feasibility, and overall comprehensiveness of the tool. The finalized version of the questionnaire is available in the Appendices. Data collectors remained available during the data collection process to clarify any questions and support complete questionnaire submission without influencing participants’ responses.

Statistical analysis

Data entry and statistical analyses were performed using IBM SPSS Statistics, version 29.0.2.0 (IBM Corp., Armonk, NY, USA). Categorical variables were summarized using frequencies and percentages, while age was summarized using the mean and standard deviation. The primary descriptive outcomes included waiting time distribution, overall satisfaction, satisfaction with waiting time, perceived quality of care, service quality indicators, and willingness to return for future urgent care services. Service quality indicators included whether patients felt their concerns were addressed effectively, whether clear instructions were provided, whether healthcare providers were perceived as knowledgeable and professional, and whether patients were informed about follow-up care. Univariate associations between categorical variables were assessed using the chi-square test. The main associations examined were overall quality of care rating with overall satisfaction, waiting time duration with overall satisfaction, waiting time duration with willingness to return for future care, and age group with satisfaction with waiting time. For inferential analysis, selected variables were collapsed into clinically meaningful categories to improve interpretability and reduce sparse cell counts. A two-tailed p-value of <0.05 was considered statistically significant.

## Results

Demographic characteristics

A total of 200 patients were included in the final analysis. The study sample was slightly male predominant, with males representing 110 participants (55.0%). The overall age profile reflected a mainly young adult population, with a mean age of 34.9 ± 12.8 years. The most represented age group was 25-34 years, followed by 18-24 years. The full demographic distribution of the participants is presented in Table 1.

**Table 1:** Demographic characteristics of study participants (N = 200)

Characteristic	Category	N (%)
Gender	Male	110 (55.0%)
	Female	90 (45.0%)
Age	18–24	44 (22.0%)
	25–34	65 (32.5%)
	35–44	41 (20.5%)
	45–54	33 (16.5%)
	55–64	13 (6.5%)
	65–80	4 (2.0%)

Reasons for visit

The presenting complaints of the 200 urgent care patients were grouped into system-based categories according to their clinical context. The most common presentations were musculoskeletal and minor orthopedic complaints, accounting for 51 patients (25.5%), followed by superficial soft-tissue injuries, 35 patients (17.5%), and gastrointestinal complaints, 34 patients (17.0%). Respiratory complaints represented 27 patients (13.5%), while cardiovascular complaints were the least frequent category, 8 patients (4.0%), as shown in Table 2.

**Table 2:** Distribution of presenting complaint categories among urgent care patients (N = 200)

Presenting complaint category	Clinical context/examples	N (%)
Musculoskeletal and minor orthopedic complaints	e.g., Back pain, joint pain, sprains, strains, suspected minor fractures, and limb pain without high-acuity features	51 (25.5%)
Superficial soft-tissue injuries	e.g., Superficial wounds, animal bites, superficial partial-thickness burns	35 (17.5%)
Gastrointestinal complaints	e.g., Mild abdominal pain, vomiting, diarrhea, dyspepsia, and constipation	34 (17.0%)
Respiratory complaints	e.g., Upper respiratory tract infection symptoms, dyspnea, asthma exacerbation and lower respiratory tract infection symptoms	27 (13.5%)
Cardiovascular complaints	e.g., Chest pain, hypertensive urgency, palpitations	8 (4.0%)
Constitutional and nonspecific complaints	e.g., Fever, fatigue, generalized body aches, and nonspecific malaise	13 (6.5%)
Miscellaneous	e.g., Vaccination, red eye, skin allergy/rash, Scheduled IV medication	32 (16.0%)

Waiting time distribution

Most patients were assessed within a short waiting period. A total of 131 patients (65.5%) were seen within 0-15 minutes, while 52 patients (26.0%) were seen within 16-30 minutes. Only 17 patients (8.5%) waited more than 30 minutes before provider assessment. Overall, this indicates that the majority of patients were assessed within 30 minutes, as represented in Table 3.

**Table 3:** Waiting time distribution among urgent care patients (N = 200)

Waiting time category	N (%)
0–15 minutes	131 (65.5%)
16–30 minutes	52 (26.0%)
31–45 minutes	10 (5.0%)
>45 minutes	7 (3.5%)

Patient satisfaction measures

Patient satisfaction measures are summarized in Table 4. Overall, most patients reported favorable satisfaction with their urgent care experience, with 183 patients (91.5%) reporting that they were satisfied or very satisfied. Satisfaction with waiting time was also high, with 167 patients (83.5%) reporting that they were satisfied or very satisfied. In addition, 125 patients (62.5%) rated the quality of care as excellent, indicating a generally positive patient perception of the urgent care service.

**Table 4:** Patient satisfaction measures among urgent care patients (N = 200)

Measure	Response	N (%)
Overall satisfaction	Very satisfied	127 (63.5%)
	Satisfied	56 (28.0%)
	Neutral	13 (6.5%)
	Dissatisfied	3 (1.5%)
	Very dissatisfied	1 (0.5%)
Waiting time satisfaction	Very satisfied	114 (57.0%)
	Satisfied	53 (26.5%)
	Neutral	21 (10.5%)
	Dissatisfied	8 (4.0%)
	Very dissatisfied	4 (2.0%)
Overall quality of care rating	Excellent	125 (62.5%)
	Very good	36 (18.0%)
	Good	13 (6.5%)
	Fair	21 (10.5%)
	Poor	5 (2.5%)

Service quality indicators

Overall, patients reported highly positive service quality indicators. Most participants felt that their concerns were addressed effectively, 193 (96.5%), received clear instructions, 195 (97.5%), and perceived healthcare providers as knowledgeable and professional, 192 (96.0%). In addition, 187 patients (93.5%) reported that they would return to the urgent care clinic for future care. However, follow-up communication was comparatively lower, as only 128 patients (64.0%) reported being informed about follow-up care. These findings suggest strong performance in communication, professionalism, and immediate care delivery, with follow-up counseling representing a potential area for improvement, as presented in Table 5.

**Table 5:** Service quality indicators among urgent care patients (N = 200)

Indicator	Response	N (%)
Concerns addressed effectively	Yes	193 (96.5%)
	No	7 (3.5%)
Clear instructions provided	Yes	195 (97.5%)
	No	5 (2.5%)
Providers perceived as knowledgeable and professional	Yes	192 (96.0%)
	No	8 (4.0%)
Informed about follow-up care	Yes	128 (64.0%)
	No	72 (36.0%)
Would return for future care	Yes	187 (93.5%)
	No	13 (6.5%)

Associations Between Patient Characteristics, Service Factors, and Urgent Care Experience Outcomes

The overall quality of care rating was significantly associated with overall satisfaction. Patients who rated the quality of care as excellent or very good were more likely to report being satisfied or very satisfied, 154 (95.7%), compared with patients who rated the quality of care as good, fair, or poor, 29 (74.4%). This association was statistically significant, χ² = 15.67, df = 1, p < 0.001, suggesting that patients’ perception of care quality was strongly related to their overall satisfaction.

Waiting time duration was also significantly associated with overall satisfaction. Among patients who were assessed within 30 minutes, 172 (94.0%) reported being satisfied or very satisfied, compared with 11 (64.7%) among those who waited more than 30 minutes. This association was statistically significant, χ² = 13.59, df = 1, p < 0.001, indicating that shorter waiting time was linked to a more favorable overall urgent care experience.

A similar pattern was observed for willingness to return for future care. Most patients who were assessed within 30 minutes reported that they would return to the urgent care clinic, 175 (95.6%), whereas this proportion was lower among patients who waited more than 30 minutes, 12 (70.6%). This association was also statistically significant, χ² = 12.19, df = 1, p < 0.001, suggesting that waiting time may influence not only immediate satisfaction but also future care-seeking preference.

In contrast, the age group was not significantly associated with satisfaction with waiting time. Satisfaction with waiting time remained generally high across age groups, including 89 (81.7%) among patients aged 18-34 years, 62 (83.8%) among those aged 35-54 years, and 16 (94.1%) among those aged ≥55 years. The association was not statistically significant, χ² = 1.67, df = 2, p = 0.435, indicating that satisfaction with waiting time did not differ meaningfully by age group, as shown in Table 6.

**Table 6:** Associations between patient characteristics, service factors, and urgent care experience outcomes (N = 200) χ²: Chi-square statistic; df: Degrees of freedom; p-value: Probability value

Association tested	Category	Outcome comparison	N (%)	χ²	df	p-value
Association between overall quality of care rating and overall satisfaction	Excellent/very good	Satisfied/very satisfied	154 (95.7%)	15.67	1	<0.001
	Excellent/very good	Neutral/dissatisfied	7 (4.3%)			
	Good/fair/poor	Satisfied/very satisfied	29 (74.4%)			
	Good/fair/poor	Neutral/dissatisfied	10 (25.6%)			
Association between waiting time duration and overall satisfaction	≤30 minutes	Satisfied/very satisfied	172 (94.0%)	13.59	1	<0.001
	≤30 minutes	Neutral/dissatisfied	11 (6.0%)			
	>30 minutes	Satisfied/very satisfied	11 (64.7%)			
	>30 minutes	Neutral/dissatisfied	6 (35.3%)			
Association between waiting time duration and willingness to return for future care	≤30 minutes	Would return: Yes	175 (95.6%)	12.19	1	<0.001
	≤30 minutes	Would return: No	8 (4.4%)			
	>30 minutes	Would return: Yes	12 (70.6%)			
	>30 minutes	Would return: No	5 (29.4%)			
Association between age group and satisfaction with waiting time	18–34 years	Satisfied/very satisfied	89 (81.7%)	1.67	2	0.435
	18–34 years	Neutral/dissatisfied	20 (18.3%)			
	35–54 years	Satisfied/very satisfied	62 (83.8%)			
	35–54 years	Neutral/dissatisfied	12 (16.2%)			
	≥55 years	Satisfied/very satisfied	16 (94.1%)			
	≥55 years	Neutral/dissatisfied	1 (5.9%)			

## Discussion

This study assessed patients’ perceptions and satisfaction with urgent care services at King Saud Medical City, a major tertiary healthcare institution within Riyadh First Health Cluster. The findings demonstrate a generally positive patient experience, with high levels of overall satisfaction, favorable perceived quality of care, short waiting times for most patients, and strong service quality indicators. Overall, these findings suggest that the urgent care clinic served as an effective care setting for adult patients with acute, lower-acuity presentations who required timely assessment and management without emergency department-level intervention.

The overall satisfaction rate in this study was high, with 183 patients (91.5%) reporting that they were satisfied or very satisfied with their urgent care experience. This finding is consistent with Riyadh-based evidence showing generally favorable patient satisfaction with urgent care clinics. In the study conducted among urgent care clinic patients at Prince Sultan Military Medical City primary healthcare centers, patients reported positive satisfaction levels, particularly in relation to provider interaction and service delivery, although areas such as waiting time, parking availability, and patient involvement in decision-making were identified as needing improvement [4]. The similarity between these findings and our study suggests that urgent care services in Riyadh are generally well perceived by patients, but that operational and communication-related aspects remain important targets for continuous quality improvement.

The pattern of presenting complaints in the present study also supports the role of urgent care clinics as an intermediate service between primary healthcare centers and emergency departments. Musculoskeletal and minor orthopedic complaints were the most common presentations, followed by superficial soft-tissue injuries, gastrointestinal complaints, miscellaneous lower-acuity complaints, and respiratory complaints. This case mix is consistent with the intended function of urgent care services, which is to provide timely care for acute but non-critical conditions. Internationally, similar acute ambulatory care models, including walk-in and GP-led urgent care centers, have been described as services designed to improve access to care, support patient flow, and reduce unnecessary pressure on emergency departments [11].

Waiting time was one of the most important operational findings in this study. Most patients were assessed promptly, with 131 patients (65.5%) seen within 15 minutes and 183 patients (91.5%) seen within 30 minutes. This likely contributed to the high overall satisfaction observed in the sample. More importantly, waiting time duration was significantly associated with overall satisfaction, as patients assessed within 30 minutes were more likely to report being satisfied or very satisfied compared with those who waited longer than 30 minutes. This finding is consistent with previous evidence showing that waiting time is a major determinant of satisfaction in urgent and emergency care settings. A study from Taif, Saudi Arabia, similarly reported that longer waiting time was associated with lower patient satisfaction in the emergency department setting [12].

The association between waiting time and willingness to return for future care is particularly important. In this study, patients assessed within 30 minutes were more likely to report that they would return to the urgent care clinic in the future compared with those who waited more than 30 minutes. This suggests that waiting time may influence not only immediate satisfaction, but also future care-seeking behavior and patient trust in the service. In addition to actual waiting time, patients’ perceptions of waiting may also be influenced by communication and transparency during the visit; a Saudi tertiary care study found that providing waiting time estimates in the emergency department was relevant to patients’ satisfaction and preference for information during waiting periods [13]. From a healthcare system perspective, this finding is important because urgent care clinics can help reduce avoidable emergency department use only when patients perceive them as accessible, timely, and reliable. A recent review of urgent care centers and emergency department demand similarly emphasized that urgent care centers may improve access and patient satisfaction when they are effectively organized and integrated into the broader healthcare system [14].

Perceived quality of care was also significantly associated with overall satisfaction. Patients who rated the quality of care as excellent or very good were more likely to report being satisfied or very satisfied overall compared with those who rated the quality of care as good, fair, or poor. This finding suggests that patient satisfaction was not determined by waiting time alone, but also by the perceived quality of the clinical encounter. This is consistent with international evidence showing that satisfaction with urgent and walk-in care is shaped by multiple factors, including access, timeliness, communication, provider interaction, reassurance, and confidence in the care received [9,11,15].

Service quality indicators were highly favorable in the present study. Most patients reported that their concerns were addressed effectively, that clear instructions were provided, and that healthcare providers were knowledgeable and professional. These findings reflect strong communication and interpersonal care, which are central components of patient-centered urgent care. Howard et al. found that patients seeking care for urgent health problems were most satisfied when care was provided by their own family physician or by an after-hours clinic affiliated with the physician’s practice, highlighting the importance of familiarity, continuity, communication, and confidence in the care pathway [9]. Although our study was conducted in a tertiary urgent care clinic rather than a family practice setting, the underlying principle is similar: patients value not only rapid access, but also reassurance, clarity, and trust in the care they receive.

Despite the generally positive findings, follow-up communication appeared to be a potential area for improvement. Only 128 patients (64.0%) reported being informed about follow-up care, which was lower than other service quality indicators. This finding is important because follow-up advice, return precautions, and safety-netting instructions are essential in urgent care settings, where patients may be discharged after a brief encounter for an acute condition. Previous studies have shown that discharge instructions are closely related to patients’ comprehension, recall, satisfaction, and safe transition after emergency care visits [16-18]. Strengthening follow-up counseling, written discharge instructions, and clear escalation advice may further improve continuity of care, patient confidence, and safety after the urgent care visit.

Age group was not significantly associated with satisfaction with waiting time in this study. Satisfaction with waiting time remained generally high across age groups, suggesting that timely assessment was perceived positively regardless of age. This finding may reflect the overall efficiency of patient flow in the clinic, where the majority of patients were assessed within 30 minutes. It may also suggest that service-related factors, particularly actual waiting duration and care organization, were more influential than demographic characteristics in shaping satisfaction with waiting time.

This study has several strengths. First, it focused specifically on urgent care services, an area with limited published evidence in Saudi Arabia, particularly within large tertiary healthcare institutions. Second, the study included a clearly defined population of adult lower-acuity urgent care patients triaged as ESI level 4 or 5. Third, data collection was conducted across different days and across three clinic shifts, which helped capture variation in patient flow and service experience. Fourth, the study assessed multiple dimensions of urgent care experience, including waiting time, overall satisfaction, quality of care rating, service quality indicators, follow-up communication, willingness to return, and factors associated with key patient experience outcomes.

Several limitations should be acknowledged. First, this was a single-center study conducted at King Saud Medical City, a large tertiary healthcare institution, which may limit the generalizability of the findings to other urgent care clinics, primary healthcare centers, private facilities, smaller district hospitals, or other regions of Saudi Arabia. Therefore, the findings and study procedures may be most directly applicable to similar high-volume tertiary care settings, while replication in smaller healthcare facilities may require contextual adaptation based on patient volume, staffing, workflow, and available resources. Second, the cross-sectional design provides a snapshot of patient perceptions during the study period and does not allow causal conclusions. Third, the sample size was determined by feasibility and patient flow rather than a formal a priori sample size calculation, which may affect the precision of some estimates and the statistical power to detect smaller associations. Fourth, random probability sampling was not used, although structured time-based recruitment across different days and shifts was used to improve sample diversity. Fifth, satisfaction measures are subjective and may be influenced by individual expectations, social desirability bias, symptom severity, or the timing of questionnaire completion. Finally, although presenting complaints were recoded into system-based categories by a clinician, some categories may still include clinically heterogeneous presentations.

Future research should include multicenter studies across urgent care clinics in different regions of Saudi Arabia to improve generalizability and allow comparison between healthcare settings. Longitudinal or repeated cross-sectional studies could also help evaluate whether patient satisfaction changes over time after workflow improvements or policy changes. In addition, qualitative studies may provide deeper insight into patients’ expectations, concerns, and suggestions for improving urgent care services. Further research should also explore the role of follow-up communication, discharge safety-netting, and integration with primary care and emergency department pathways in improving patient experience and continuity of care.

## Conclusions

This study provides a focused evaluation of patients’ perceptions and satisfaction with urgent care services at King Saud Medical City, Riyadh, Saudi Arabia. The findings demonstrated a generally positive patient experience, with high overall satisfaction, favorable perceived quality of care, and strong service quality indicators. Most patients were assessed within 30 minutes and reported that their concerns were addressed effectively, clear instructions were provided, and healthcare providers were knowledgeable and professional. Waiting time and perceived quality of care were significantly associated with patient experience, as patients assessed within 30 minutes and those who rated care as excellent or very good were more likely to report overall satisfaction and willingness to return for future care. In contrast, age group was not significantly associated with satisfaction with waiting time, suggesting that service-related factors may have a greater influence on patient experience than demographic characteristics alone.

Despite the overall favorable findings, follow-up communication emerged as a potential area for improvement, as fewer patients reported being informed about follow-up care compared with other service quality indicators. Strengthening discharge counseling, follow-up instructions, and safety-netting advice may help improve continuity of care and patient confidence after urgent care visits. Future multicenter studies across different urgent care settings in Saudi Arabia are recommended to enhance generalizability, compare service models, and guide patient-centered quality improvement initiatives.

## Appendices

Questionnaire

Demographic Characteristics

Gender:

Male

Female

Age group:

18-24 years

25-34 years

35-44 years

45-54 years

55-64 years

65-80 years

Reason for Urgent Care Visit

What was the main reason for your urgent care visit today? 

This question asked about the patient’s main presenting complaint. The responses were later classified into categories such as:

-Musculoskeletal and minor orthopedic complaints

-Superficial soft-tissue injuries

-Gastrointestinal complaints

-Respiratory complaints

-Cardiovascular complaints

-Constitutional and nonspecific complaints

-Miscellaneous

Waiting Time

How long did you wait from triage until being assessed by a healthcare provider?

0-15 minutes

16-30 minutes

31-45 minutes

More than 45 minutes

Overall Satisfaction

Overall, how satisfied were you with your urgent care visit today?

Very satisfied

Satisfied

Neutral

Dissatisfied

Very dissatisfied

Satisfaction With Waiting Time

How satisfied were you with the waiting time before being assessed by a healthcare provider?

Very satisfied

Satisfied

Neutral

Dissatisfied

Very dissatisfied

Overall Quality of Care Rating

How would you rate the overall quality of care you received today?

Excellent

Very good

Good

Fair

Poor

Service Quality Indicators

Were your concerns addressed effectively?

Yes

No

Were clear instructions provided?

Yes

No

Were healthcare providers knowledgeable and professional?

Yes

No

Were you informed about follow-up care?

Yes

No

Would you return to this urgent care clinic for future care?

Yes

No
